# Leading the digitalisation process in K–12 schools – The school leaders’ perspective

**DOI:** 10.1007/s10639-023-11935-x

**Published:** 2023-06-10

**Authors:** Jussara Reis-Andersson

**Affiliations:** Department of Education, Mid Sweden University, Sundsvall, Sweden

**Keywords:** Digital competence, Digitalisation, K–12 education, K–12 schools, Leadership for digitalisation, School leader

## Abstract

Digital technologies have increased worldwide in the last years. In addition, the pandemic has strengthened digital technologies’ roles in education, requiring twenty-first-century skills, such as digital competence, and indicating a new normal. Digitalisation in education creates opportunities that could lead to positive effects if digital technologies are applied correctly. However, applying digital technologies can incorrectly trigger a negative development – for example, increasing workload due to nonfriendly user interfaces in software and reducing the motivation to apply digital technologies in education due to a lack of digital competence. Teachers require access to digital technologies and digital competence related to educational issues that influence equality within and among K–12 schools, thus making school leaders’ roles crucial in digitalising education. Three group interviews and a survey were used to collect the data in a network of three municipalities in Sweden. The data have been categorised and analysed with thematic analysis. The results show that school leaders describe the digitalisation process in the form of digital competence for teachers, access to hardware and software, and a shared culture. School leaders explain that clear guidelines, collaboration between teachers, and enough time enable digitalisation in education. However, the lack of support and the lack of resources constrain digitalisation in education. At the same time, school leaders do not often discuss their own digital competence. The school leaders’ roles in the digitalisation of K–12 schools are important, requiring digital competence for leading the digitalisation process.

## Introduction

Digitalisation creates changes in society that increase the demand for different professional sectors, and these changes require new skills – often twenty-first-century skills such as digital competence – that individuals should bring with them into their lives. K–12 schools have a central role in developing society and preparing young people for an uncertain future, specifically regarding which new digital technologies will emerge in the following decade. Digitalisation in K–12 schools is nothing new; however, the need to use digital technologies during the COVID-19 pandemic clarified the opportunities and challenges digital technologies bring (Corlatean, 2020; Khlaif et al., 2021), influencing digital technologies’ roles in education. The pandemic has generated challenges, such as skills to use digital technologies in teaching and unexpected opportunities (Corlatean, 2020), changing the normal in the education system. Producing new materials for teaching and collaboration in K–12 schools are some examples of opportunities with digital technologies in education (Corlatean, 2020; Uzorka & Olaniyan, 2022).

According to the Organisation for Economic Co-operation Development (OECD, 2021), digital technologies affect several facets of education, providing students with new opportunities to learn in and outside of schools and changing pedagogical approaches and students’ learning experiences. In 2017, the Swedish Government (2017) developed a digital strategy plan for the sustainable expansion of digital technologies in education to meet this digital society’s needs. Gu and Lindberg (2021) pointed out that the latest Swedish digitalisation strategy was aimed at increasing the importance of using digital technologies to gain knowledge and achieve equality. However, steering, organising, and leading the expansion of digital technologies in K–12 schools require school leadership, which is crucial for expanding digital technologies (Fullan, 2015; Hauge et al., 2014; Leithwood et al., 2019).

School leaders are responsible for creating conditions for teachers to apply digital technologies in education. Nevertheless, these conditions depend on the rapidly developing digital technologies (Olofsson et al., 2020), they create challenges in schools (Masters, 2018), and they require support in the act of leading and teaching (Håkansson Lindqvist & Pettersson, 2019; Reis-Andersson, 2023). A successful component in creating possibilities for expanding digital technologies in education is how leaders lead this work (Agélii Genlott et al., 2021). Liljenberg (2015) explained that “leadership is considered to be significant for creating a developing and learning school organization” (p. 152).

Håkansson Lindqvist (2019) stressed that applying digital technologies in schools continued to increase and that it was the school leader’s responsibility to create conditions for teachers. How digital technologies are implemented and prioritised in schools may be connected to the importance of grades and effectiveness–for example, students’ results and teachers’ interest in applying digital technologies in teaching. However, the implementation work is often not apparent; technology is a secondary element and usually something that “individual staff could take a personal lead on if they so wished” (Selwyn et al., 2018, p. 54). Even Haelermans (2017) highlighted that the way digital technologies were implemented and applied in education influenced how effective these technologies would be in learning situations, requiring priority and attitude towards implementation work from school leaders’ and teachers’ perspectives. The scholar highlights that the human factor’s role, for example, “teachers’ and school leaders’ negative beliefs and attitudes” (Haelermans, 2017, p. 24) towards digital technologies are important and should not be underestimated. Håkansson Lindqvist (2019) emphasised the importance of school organisers in supporting school leaders for “how school leaders’ practices support, promote, and advance innovative and sustainable teaching and learning environments through the use of digital technologies” (p. 1226). However, information on how municipality school leaders organise digital technologies in their schools is lacking, and more research is needed (Christensen et al., 2018; Håkansson Lindqvist, 2019; Pettersson, 2021). This paper contributes to the growing research on the digitalisation process in K–12 schools from the school leaders’ perspective.

- How do municipality school leaders describe the digitalisation process in K–12 schools?
- What enables and constrains the digitalisation process in municipality K–12 schools?

## Background

Digital technologies in K–12 schools are not new. However, they have increased in the last few years, especially during the pandemic, creating opportunities for networks and challenges related to financing digital technologies in schools (Reis-Andersson, 2023). Moreover, digital technologies shape what digital competence and twenty-first-century skills people need to develop, indicating a new normal, for example, the opportunity for online participation (Jandrić et al., 2022). In Sweden, the government developed a digitalisation strategy for 2017 to digitalise K–12 schools. From this, the government expected that all students would develop digital competence and that digitalisation opportunities in education would be utilised to improve students’ results. School leaders are responsible for creating opportunities for teachers to apply digital technologies in teaching. According to Ewing and Cooper (2021), school leaders are crucial in supporting teachers and establishing a culture that enables applying digital technologies in education. At the same time, Agélii Genlott et al. (2019) pointed out that applying digital technologies required changes in education. Howard et al. (2021) claimed that “institutional responses at times of rapid changes in teaching and learning could possibly compensate for lack of time and/or training for teachers to appropriately prepare for changes” (p. 154). In effect, school leaders and teachers need time and support regarding training to develop their digital competence.

Researchers have indicated that expanding digital technologies in K–12 schools requires competence to organise and lead the digitalisation process. Expanding digital technologies in education is a complex process that requires strategic planning and a commitment to success (Hopkins, 2017). New digital technologies develop rapidly, leading to changes and a need for supporting teachers. Applying digital technologies in teachers’ daily practice requires digital competence, which is an organisational issue (Agélii Genlott et al., 2021). However, according to Pettersson (2021), digitalisation processes require pedagogical and organisational changes, influencing how digital technologies are applied in education. Viberg et al. (2020) emphasised that expanding the access to and application of digital technologies in classrooms does not automatically lead to improvement in teaching. It is also about how digital technology is applied if the aim is to benefit students’ learning. It requires an understanding of what enables and constrains (Kemmis et al., 2014) the organisation of digital technologies in K–12 schools.

Conrads et al. (2017) highlighted that an important key to embedding technological innovation into teaching is appointing school leaders that create conditions for their teachers. Leadership is crucial for an efficient digitalisation process, but even time and other resources are important to facilitate the change process. At the same time, school leaders shape the digitalisation process from top-down policies, which can contradict how teachers want to integrate digital technologies in their classrooms. Woodcock and Hardy (2022) investigated school leaders’ perceptions of both policy and practice and showed that policies were characterised by complexity and nebulous conceptions that may not match school leaders’ desires. For example, disinformation in social media may create a desire for leaders that act at once instead of first gathering knowledge to make decisions (Jandrić et al., 2020).

### Digitalisation in schools

Digital technologies are also very much about resources, which can create inequality within and among schools. Organising digital technologies in schools requires support and digital competence. Reis-Andersson (2023) explained that digitalisation in schools, for example, was about “putting the right competence in the right place” (pp. 80–81), reducing costs in that way. However, how school leaders organise the expansion of digital technologies in education may differ from one school to another because the schools have different conditions (Håkansson Lindqvist & Pettersson, 2019). In addition, various age groups may have different needs. For example, a preschool’s needs for digital technologies may differ from a compulsory school and an upper secondary school.

Schools that are successful in the development work are usually better at handling change, which Larsson and Löwstedt (2020) defined as school leaders’ competence to organise for changes. School development demands courage and requires school leaders’ encouragement. Technology integration is not yet achieved systematically in most schools; according to Tondeur et al. (2017), it requires organising and leading competencies and understanding how digital technologies influence education and students’ lives.

### Leading the digitalisation process

School leaders need to understand how to organise digital technologies in schools and how to sustain innovation processes by understanding change processes (Agélii Genlott et al., 2021) and sharing responsibilities. It is an issue of digital competence (Håkansson Lindqvist, 2019; Håkansson Lindqvist & Pettersson, 2019) and a lifelong learning perspective (Jaldemark et al., 2021) for school leaders. Jaldemark et al. (2021) pointed out that “digitalisation is a pronounced societal trend impacting on lifelong learning and lifelong education” (p. 1578), which influences school leaders’ attitudes towards digital technologies and thereby enabling and constraining (Kemmis et al., 2014) the digitalisation process. School leaders are also responsible for teachers’ continuing professional development and the teachers’ opportunities to increase their professional competence. Mingaine (2013) pointed out that school leaders’ behaviour and positive attitudes towards implementing digital technologies were important to involve and activate teachers in the digitalisation process. Even Reis-Andersson (2023) emphasised the importance of school leaders’ attitudes towards teachers to give legitimacy to the digitalisation process. The scholars explained how the municipal school organiser prioritises the expansion of digital technologies in K–12 schools will influence the school leaders’ attitudes to the digitalisation process and thereby the teachers’ view of applying digital technologies.

Agélii Genlott et al. (2021) investigated challenges in leading digital technologies in education and stressed that solutions for the K–12 schools challenges must be spread, enabling the digitalisation process. Moreover, these scholars stressed that teachers should be able to improve their collaborative working methods in addition to their teaching. It requires school leaders to want to achieve changes in the digitalisation process. Spreading solutions for digital technologies in education is a school leader’s issue that requires them to “engage with research and innovation so as to be able to see the difference between the latest fad and a seed of improved practice” (Agélii Genlott et al., 2019, p. 3034). At the same time, Ittner et al. (2019) pointed out that making changes in schools required innovation and organisational resources.

School leaders find it challenging to support teachers in applying digital technologies in education based on the curriculum (AlAjmi, 2022; Flanagan & Jacobsen, 2003; Pettersson, 2018). Many school leaders “have not been prepared for their new role as technology leaders, and have therefore struggled to develop both the human and technical resources necessary to achieve ICT [information and communications technology] outcomes in their schools” (Flanagan & Jacobsen, 2003, p. 127). Nevertheless, school leaders are crucial to expanding digital technologies in K–12 schools (A’mar & Eleyan, 2022; Raman et al., 2019).

Increased access to digital technologies in society places demands on new skills for individuals. K–12 schools must prepare students for the opportunities and challenges digital technologies bring. However, the digitalisation process in schools requires organisation. School leaders must lead and organise digital technologies in their schools. Considering this, the study further explores the school leaders’ roles in leading and organising digital technologies in K–12 schools.

## Method

The data were collected in a network of three municipalities in Sweden, denoted as Municipalities A, B, and C. The research was conducted in public municipality K–12 schools, and data were collected from public municipality school leaders. These municipalities have different sizes and numbers of schools. The biggest municipality has about 100,000 citizens and 77 preschools, 25 compulsory schools, and two upper secondary schools. The second municipality has about 25,000 citizens, 17 preschools, 13 compulsory schools, and one upper secondary school. Finally, the smallest municipality has about 18,000 citizens and 13 preschools, nine compulsory schools, and one upper secondary school.

A survey was conducted with school leaders from municipalities A, B, and C, representing all levels from preschool to upper secondary school. Cohen et al. (2011) pointed out that surveys could be used to measure or generalise but also “to catch local, institutional or small-scale factors and variable – to portray the specificity of a situation” (p. 257), which is the case in this study. The survey’s questions were based on discussions conducted at five municipality network meetings between November 2018 and February 2021. Two of these meetings were conducted via video conferencing due to the pandemic. During the meetings, school organisers from the three municipalities shared their knowledge and experiences related to the digitalisation process in their schools. They expressed what activities they had done, are doing, or planned to do to increase digital competence and access to digital technologies in their K–12 schools based on the national digitalisation strategy, their digitalisation plans, the current situation, and a needs analysis. However, the municipalities have different contextual conditions, such as values, structures, and resources, which must be considered. This study provides an understanding of aspects of the digitalisation process in public municipality K–12 schools from the school leaders’ perspective. It also can be applied in municipalities with similar contextual conditions. In total, 101 pages of transcriptions, meeting protocols, and notes were produced. The data from these meetings and the municipalities’ documents, such as digitalisation plans, situation analyses, and needs analyses, were the basis for constructing the survey for the school leaders. For example, the survey had 15 questions. It was constructed with six background questions about the respondents, five open questions, and four questions with 20 statements on a 5-point Likert scale about digitalisation in schools. However, only the open questions have been used in this paper.

A pilot study was completed to sharpen the questions in the survey. After that, the survey was revised and sent via email to 157 school leaders in Municipalities A, B, and C: 38 school leaders for preschools, 88 school leaders for compulsory schools, and 31 school leaders for upper secondary schools. The survey was self-administered and sent to the school leaders’ email addresses. It was open between June 2021 and October 2021. About three reminders each month were sent during this time. Ninety-six school leaders answered the survey – 32 school leaders for preschools, 50 school leaders for compulsory schools, and 14 school leaders for upper secondary schools – corresponding to about 60% of the respondents.

Data were collected in three semistructured group interviews, one for each participating municipality. Three areas were used for the group interview questions: school leaders’ collaboration with school organisers, the type of actions and changes for expanding the access to and application of digital technologies in education, and what enables and constrains the digitalisation process. The questions for the areas in the group interviews were based on data collected from the network meetings and this paper’s research questions.

Group interview participants had worked between five to 20 years as school leaders. Four school leaders of the same municipality represented different school levels in each group interview. For example, one school leader represented preschools (students aged 0–5 years old); two school leaders represented compulsory schools, including years 0–6 (students aged 6–12 years old) and years 7–9 (students aged 13–15 years old); and one school leader represented upper secondary schools (students aged 16–18 years old). Unfortunately, just before the group interview, one school leader announced that due to unpredictable events at school, this school leader could not participate in the group interview. Therefore, two of the three group interviews were conducted with four school leaders. One interview was conducted with three school leaders. The school leaders in the group interviews represented preschool to upper secondary school. Their answers may be very different depending on which level they represent. Even preschools and schools of the same level differ depending on the local contextual conditions. However, this study strived to find points of intersections between preschool to upper secondary school.

Each interview was conducted via video conferencing for 60 to 90 min. In total, the interviews generated 109 pages of transcriptions. Cohen et al. (2011) argued that a group interview “can generate a wider range of responses than in individual interviews” (p. 432). The scholars also stressed that group interviews saved time because they were often quicker than individual interviews, which is a practical and organisational advantage. However, conducting group interviews during the pandemic time implied some challenges; for example, it was difficult to find school leaders for the group interviews, and school leaders needed to prioritise their time due to, for instance, sick leave in schools. In the end, 11 school leaders participated in the three group interviews. In addition, conducting interviews via video conferencing makes it difficult to capture school leaders’ body language, emotions, and behaviours, which may be important if the school leader is uncomfortable with a specific question.

The thematic analysis used to categorise the collected data included a six-phase framework: (1) familiarisation with the data, (2) generate codes, (3) search for themes, (4) review themes, (5) define themes, and (6) write-up (Braun & Clarke, 2021). In the first phase, I familiarised myself with the data by reading and rereading the transcripts and making notes. I organised the data systematically in the second phase to generate the initial codes. I had the research questions in mind, which influenced the thematic analysis to a more theoretical than inductive approach. Braun and Clarke (2021) explained that the coding was founded in the data set, but the research questions and the provided codes reflected theoretical ideas that the researcher hoped to strengthen understanding through the data set. In the third phase, themes have been constructed, thus sharing meaning. Braun and Clarke (2006) pointed out that the rules for making a theme were not fast, but a theme needed to capture something significant relating to the research questions. In the fourth and fifth phases, the themes were reviewed and combined, forming finally three themes and 15 subthemes connected to actions, enabling, and constraints concerning the digitalisation process. In the last phase, the report was produced, and some quotes were used to support the themes and subthemes.

## Results

This section presents how school leaders describe the digitalisation process in municipality K–12 schools and what enables and constrains this digitalisation process. Three themes have been constructed: actions that support the digitalisation process in K–12 schools, enabling the digitalisation process in K–12 schools, and constraining the digitalisation process in K–12 schools. At the end of this section, Table 1 summarises the results of this paper.

**Table 1:** Themes and subthemes

Actions that support the digitalisation process in K–12 schools	Enabling the digitalisation process in K–12 schools	Constraining the digitalisation process in K–12 schools
Increasing teachers’ digital competence by attending courses, participating in workshops, collegial learning, and networking.	Positive attitudes towards technology enable the digitalisation process in education.	School leaders and teachers’ lack of digital competence.
Investing in hardware and software.	Opportunities to differentiate education provide conditions for increasing the quality of teaching.	Schools lack access to resources, or they have financial limitations.
Supporting teachers’ work by applying digital technologies in teaching.	IT strategists act as a “spider on the web” and liaise between school organisers and school leaders.	Mainstream schools and schools for students with special needs lack support.
Creating a culture in which digital technologies permeate teaching.	Strong relationships permit clear communication among school leaders, school organisers, and the IT department.	Attitudes to digital technology may slow the digitalisation process in education.
Changing methods in both teaching and administration.	Clear guidelines, plans, and budgets ensure that authority in schools becomes more legally secure.	The technological infrastructure is nonfunctional.

### Digital competence and attitudes towards digital technology

Working to increase *digital competence* in schools is important, and Municipality B described that “we have some internal training for software that we have bought for the schools” (B, survey, 2021) as activities for increasing digital competence in public municipality schools. Municipality B pointed out that workshops among the teachers were carried out in 2021. Increasing teachers’ digital competence is a way to influence teachers’ attitudes toward working with digital technologies. In addition, students should be involved in using digital technologies in different ways, and they should “be a part of the work” (C, group interview, 2021). Municipality B stressed that teachers must work with source criticism in their schools, making students aware of the opportunities and challenges digital technologies bring. They also highlighted the need to make students producers and not just consumers of digital technologies so that they can create texts, videos, sounds, and pictures. According to Municipality A, “there is nobody responsible for increasing digital competence in schools, nobody on the school organiser level” (A, group interview, 2021).

School leaders’ *lack of digital competence* influences teachers’ digital competence and K–12 schools’ digitalisation process. According to the municipalities, there is a need for teachers’ digital competence. At the same time, Municipality A pointed out that it was a financial issue; thus, it was expensive to send teachers to courses. Municipality C emphasised that “digital competence in schools needs to be increased for the teachers to be confident in applying digital technologies in their teaching” (C, survey, 2021). Municipality A described the school organiser’s digital competence as low, with anyone who has something to say steering it. Municipality A also emphasised that “we must dare to look at what level of digital competence we have because it can be a problem” (A, group interview, 2021). They stressed that school organisers’ low digital competence might influence the support for school leaders and, therefore, constrain the digitalisation process in schools. Both Municipalities B and C have worked with teachers’ *collegial learning* for the last few years. They described teachers’ collegial learning as a way to increase their digital competence and establish a shared culture. “We have created a digitalisation group and worked with the teachers’ lesson tips and education efforts” (C, survey, 2021). Municipality B highlighted that “everyone in the work team participated in the same training contributes to an increasing degree of agreement” (B, survey, 2021).

### Investing in hardware and software and opportunities for financing in K–12 schools

The municipalities have invested in digital technologies in education. However, more *hardware* and *software,* such as computers and tablets, are needed in K–12 schools. Municipality B stressed that they “have purchased Chromebook” (B, survey, 2021), and Municipality C emphasised that “all students have been given access to a Chromebook” (C, Survey, 2021). When hardware and software are purchased, it is important to have clear instructions on how a specific digital technology should be used and maintained, for example, before handing it in for summer storage. In another way, there is a risk that it will not work in the teaching situation – for example, when a whole lesson requires access to a computer or Chromebook. Then, using digital technologies may be a challenge instead of an opportunity. Challenges that arise during the digitalisation process must be addressed because they affect teaching, but “sometimes it is difficult to reach [to achieve a solution]” (C, group interview, 2021). Implementing new digital technologies creates opportunities and challenges for the various forms of education. However, decisions (e.g., purchasing hardware and software) should be correct when the conditions are known. Municipality C claimed that “the decision is wrong when you know what prerequisites you have, yet the decision cannot meet the prerequisites” (C, group interview, 2021). Software is widely used; it is “purchased, and teachers have started using software to varying degrees” (C, survey, 2021). According to Municipality B, using video conferencing for meetings with teachers, students, parents, and other stakeholders and utilising cloud services have increased due to the pandemic. They have also implemented possibilities for people to participate in digital field trips. These activities “have developed our digital competence” (B, survey, 2021).

Although access to digital technology is an enabler in education, the *lack of resources* is a constraint for schools, creating challenges regarding the access to and application of digital technologies in education. School leaders emphasised that it was about resources and argued that it would be a good investment as working methods become more effective. Even Municipality B stressed the lack of resources as a constraint: “There are too few financial resources, which means that we cannot use digital technologies to the extent we want” (B, group interview, 2021) – for example, access to digital teaching materials, such as digital learning books, tablets, and computers. They pointed out that finances made it difficult for a school to buy digital and hardcopy literature. Municipality B explained that they must rent expensive hardware, “which erodes the financial space for teaching materials and teachers” (B, group interview, 2021). The school leaders also pointed out that they had precise requirements but no funds set aside for development or trying something new: “We apply for government grants insofar as possible” (B, group interview, 2021).

### Supporting the digitalisation process of K–12 schools and IT strategists

Teachers also need support to embrace digital technologies in their teaching. School leaders’ support for teachers is key to expanding digital technologies in education. At the same time, school leaders need support in this work. A *lack of support* may constrain the digitalisation process in schools. School organisers are important to ensure school leaders’ success in increasing teachers’ interest in applying digital technologies in their teaching. Municipality A pointed out that “unfortunately, support for the schools is minimal” (A, group interview, 2021). Municipality B also highlighted that digital technologies might increase flexibility. Still, there is much administration, increasing the need for support: “Everything has to be maintained, and there is not always competence for it in every school” (B, survey, 2021). They also pointed out that “clarity in where to turn in the current situation can enable or constrain the digitalisation process” (B, group interview, 2021) and that “sometimes you do not know where to turn to” (C, group interview, 2021). They also emphasised that support could be costly for schools.

*For students with special needs*, digital technologies are important; at the same time, school leaders pointed out that there was “a lack of support and knowledge about both special software and hardware used in schools” (B, survey, 2021). According to Municipality C, “more support is needed, and the need for support looks different” (C, group interview, 2021) for all age groups. Teachers must solve the problems even though digital competence is lacking. Another challenge is limited licences for software for students with special needs, leading to an increased cost for schools “based on the fact that there is limited software that we have, for example, a municipality licence for” (B, survey, 2021). The school leaders stressed that digital technologies could be used to benefit the communication and activity of students with special needs, for example, students who need digital technology to communicate with others. Still, there is a need for digital competence both at the municipality level and at K–12 school level so that school leaders can take part fully in the range of available digital technologies: “Several times, we have been stuck in technological challenges, and we may contact the manufacturer, but they may not always be able to help because a firewall has been set up” (B, survey, 2021). The school leader argued that it would be most valuable if the information technology (IT) department had knowledge about schools for students with special needs.

### Relationship and communication in the digitalisation process of K–12 schools, creating a culture in which digital technologies permeate teaching

From the school leaders’ perspectives, another resource that could enable or constrain the digitalisation process in education is *IT strategists* who have knowledge of and experience with both pedagogy and technology. In addition, by representing the school organiser, these strategists link school leaders, school organisers, and the IT department. One school leader recognised the value of this liaison, saying, “it has been successful in having someone who has been the spider in the web, bringing the technological and pedagogical issues together” (C, group interview, 2021). Good *communication* between school leaders and teachers and school leaders and between school organisers is important, including communication in how school leaders and teachers use pedagogical concepts regarding digital technologies. A school leader from Municipality B emphasised, “we have had an active dialogue with the school organiser” and “we are often in contact with the IT department based on digital development” (B, group interview, 2021). *Collaboration* between school leaders and school organisers and among K–12 schools and IT departments is important to expand access to and the application of digital technologies in schools. Municipality B’s school leader pointed out that the collaboration with the school organiser and the IT department had “worked well the last years” (B, group interview, 2021). At the same time, this school leader pointed out that the number of employees at the manager and school level had reduced, leading to difficulty in dialogue with the school organiser and affecting the schools’ digitalisation process. However, even Municipality A’s school leader pointed out the collaboration with the school organiser had been effective. Digitalisation ensures that authority in schools becomes more *legally secure* because much of the school documentation is done digitally, according to a leader from Municipality C. In addition, a leader from Municipality B indicated that digital technologies saved time by making their work smoother and more straightforward and that the technologies had made contact with the school organiser faster and more harmonious. Digital technologies produce *opportunities to differentiate* education support and develop creativity. It is also possible to increase equality when the conditions of digital technology implementation are the same across schools.

### Changing methods in both teaching and administration in K–12 schools’ digitalisation process, clear guidelines, plans, and budgets

A *functional infrastructure* enables the digitalisation process. The digitalisation of education “must be run by the school organiser” (A, group interview, 2021) with clear guidelines and a clear plan, focusing on the local level. The digitalisation process also must be evaluated and analysed. School leaders emphasised that because “digitalisation is costly in schools,” it requires “a long-term plan” (C, group interview, 2021) and budget to increase equality within and among schools. The school leaders pointed out that often the school organiser was responsible for funding the significant investments the expansion of digital technologies demanded, such as software. However, the schools “need to buy more specific digital technologies adapted for children’s needs” (A, group interview, 2021). Because these costs influence the school’s budget, they may influence equality within and among schools. The need for sustainable, functional infrastructure may be challenging to meet. There is a lack of equality among schools; specifically, “access to digital technologies varies greatly among schools” (B, survey, 2021). After implementing digital technologies, schools have struggled with challenges such as Chromebooks’ unsuitability for certain software, lack of software for students with special needs, and limits on recording videos, taking photos, and saving these videos and pictures.

School leaders also mentioned “regular training, surveys, and networking” (A, survey, 2021) as ways to continue the digitalisation process. However, the misapplication of digital technologies in education may generate negative effects. For example, nonfriendly user interfaces in software may increase users’ workloads and reduce their motivation to use digital technologies in education due to their self-perceived lack of digital competence. According to one of Municipality A’s leaders, the school “is strangely ancient in terms of technology” (A, survey, 2021). However, pandemic-era instruction may have provided teachers the sense that their work was digitalised through the use of cloud services and distance education. In this case, this sense can create a need to understand what the digitalisation process entails, according to Municipality B. Municipality C’s school leader pointed out that certain solutions (for example, distance education) that the pandemic’s circumstances necessitated may have led some teachers to feel that their teaching was sufficiently digitalised, causing their reluctance to continue the digitalisation process. This leader noted, “it is important to reflect on digital teaching and how it looks” (C, group interview, 2021).

Digitalisation provides conditions for increasing the quality of education, for example, through more flexible teaching. Teaching materials are handled digitally, giving students the possibility to access those materials at any time. Students also can participate in distance education, which proved effective during the pandemic. Furthermore, digitalisation increases possibilities for teachers to collaborate “through collaboration platforms and shared planning documents as well as access for continuing education no matter where you [teachers] are” (B, survey, 2021). Access to digital technologies is “important in achieving equality because not all students have access to digital technologies at home” (B, survey, 2021). With digital technologies, schools can reach more students and teach them in a more accessible way. Digitalisation also can provide higher goal fulfilment for students in the long run.

Table 1 summarises this paper’s results and identifies their relevance to the research questions.

Table 1 presents a brief overview of actions that support the expansion of digital technologies in K–12 schools and what enabled and constrained these actions. The subthemes presented overlap with each other, making it difficult to present based on a certain order. For example, positive attitudes towards technology reflect not only in creating a culture in which digital technologies permeate teaching but also in how much school leaders invest in hardware and software, invest in teachers’ digital competence, and support teachers’ daily work in applying digital technologies in teaching. In addition, financial limitations influence the school leaders’ possibilities to invest in hardware, software, and activities to increase teachers’ digital competence. In particular, the results show that digitalisation demands change, creating several educational opportunities and challenges.

## Discussion and conclusions

The first research question was how school leaders describe the digitalisation process in K–12 schools. The results show that school leaders build digital competence through workshops, internal training, and collegial learning. However, although teachers use several digital technologies, many of their working methods have not changed, according to the school leaders, creating a need for digital competence, built in an ongoing learning process that is undertaken with a lifelong learning perspective (Jaldemark et al., 2021) and is engaged in individually, in groups, and across a shared culture. The school leaders stated that development also took place in a school through collegial learning. At the same time, the school leaders highlighted that communication between teachers significantly influenced how schools built and developed a shared culture, which is an important issue for school leaders (Ewing & Cooper, 2021).

School leaders need to prepare teachers to apply digital technologies in teaching. The teacher’s responsibility is to organise, plan, and implement teaching that enables students to use digital technologies to develop their knowledge. Teachers must understand not only which digital technologies should be applied in the classroom but also how and why to apply these technologies (Haelermans, 2017). Therefore, teachers need a defined pedagogical perspective as a starting point for understanding the reasons for and methods of applying digital technologies in their teaching, requiring organised changes in education (Agélii Genlott et al., 2019; Agélii Genlott et al., 2021). In light of this, digital competence is important at all levels of the chain of command, even for school organisers and school leaders. School leaders need digital competence to organise and lead technology implementation in schools (Håkansson Lindqvist, 2019; Håkansson Lindqvist & Pettersson, 2019; Pettersson, 2021). The organisation of schools should be based on the school’s needs and conditions (Håkansson Lindqvist & Pettersson, 2019). In other words, school leaders’ digital competence is important for the digitalisation process in K–12 schools. School leaders may increase their digital competence by collaborating and sharing knowledge and experiences with each other in a network that includes school organisers.

Even though digital technologies have expanded in education in recent years, especially concentrated around the pandemic, there is a need for more adapted digital technologies in various age groups, according to the school leaders in this study. For example, digital technologies in preschool may be used differently than in compulsory or upper secondary school. Haelermans (2017) emphasised that teachers needed time to learn how to apply specific hardware or software in teaching or how to use a specific system or platform, which school leaders could support by preparing for changes (Howard et al., 2021; Larsson & Löwstedt, 2020) and strategic planning (Hopkins, 2017). Furthermore, school leaders and teachers need to create a common consensus on how they should use the pedagogical concepts that influence equality within and among schools. However, school leaders’ and even school organisers’ attitudes towards digital technologies influence teachers’ attitudes towards the practical application of these technologies, as Reis-Andersson (2023) and Mingaine (2013) confirmed. Therefore, school leaders should encourage teachers to apply digital technologies in teaching and they should support teachers by emphasising how such technologies may be applied (Viberg et al., 2020).

The second research question concerned what enables or constrains the digitalisation process in the municipalities’ K–12 schools. According to school leaders, strong relationships across the chain of command and with the IT department importantly enable digitalisation and provide opportunities to increase equality within and among a municipality K–12 schools. As Gu and Lindberg (2021) pointed out, access to and the application of digital technologies are connected to equality in schools, thereby affecting education’s role in society. School organisers and school leaders must create opportunities and conditions in which teachers can access and apply digital technologies in their teaching. By collaborating with each other; sharing knowledge, experiences, methods, and examples; and connecting through IT strategists, school organisers, and school leaders work together to create an organisation that facilitates access to and the application of digital technologies in their teaching. According to Municipality A’s school leaders, the guidelines, plans, support, and resources enable their schools’ digitalisation process. However, school leaders should draw on their base of digitalisation knowledge and provide strong leadership simultaneously (Jandrić et al., 2020), clarifying expectations and attitudes towards the process of digitalisation in education. They also need to be aware of and understand the policy they are implementing (Woodcock & Hardy, 2022), creating conditions for teachers (Conrads et al., 2017) to apply digital technologies in teaching. Organising digital technologies in K–12 schools requires digital competence (Agélii Genlott et al., 2021; Håkansson Lindqvist, 2019; Håkansson Lindqvist & Pettersson, 2019) to understand how digital technologies affect students’ lives and the opportunities and challenges digital technologies bring in education.

School leaders emphasised the importance of using digital technologies in schools for students with special needs. Still, they pointed out a lack of support and knowledge about software and hardware for students with special needs. Even teachers’ lack of digital competence and limited licenses for software at the municipality level are challenges for these school leaders; often, these licenses are issued at the municipality level. Therefore, these challenges confirm the importance of school leaders’ understanding of their new role as technology leaders (Flanagan & Jacobsen, 2003). A’mar and Eleyan (2022) and Raman et al. (2019) emphasised the importance of leadership in the process of digitalisation in education and of leaders’ understanding of the changes that digitalisation brings (Ittner et al., 2019). At the same time, it is important to be aware that using digital technologies in teaching can change teachers’ roles (Tondeur et al., 2017). School leaders highlighted the many possibilities of digital technologies in schools and emphasised concepts, such as improvement and development, when they discussed those technologies’ expanded application in teaching. At the same time, the leaders identified teachers’ lack of digital competence, support, and resources dedicated to the digitalisation process in schools, which Håkansson Lindqvist (2019) and Håkansson Lindqvist and Pettersson (2019) emphasised was crucial for the progress of digitalisation in education. Therefore, digital technologies are an enabler of change in education, requiring organisational support and leadership. However, applying digital technologies may also be challenging for school leaders and teachers if the organisation and leadership are unclear.

Consequently, large investments are made at the municipal level, but specific purchases for some schools are made based on the school’s budget. The school’s financial situation can enable or constrain teachers’ digital competence, for example, by providing competence-development initiatives or time for continuing education and access to hardware and software. Access to support enables applying digital technologies in education, hopefully leading to fewer technology challenges in classrooms. Existing competence in the schools must be used in the right way, which Reis-Andersson (2023) also highlighted. Opportunities for purchasing the right digital technology and using the competence in schools the right way, getting access to digital competence, and support for applying digital technologies in teaching are challenges that many schools, independent of conditions, need help with.

In summary, the school leaders’ capacity to lead the digitalisation process in education should begin with a holistic perspective that establishes the golden thread of their schools’ digitalisation. Leaders should be able to understand the opportunities and challenges that digital technologies bring to education. However, an interesting point in this study is that school leaders did not often reflect on or discuss their own digital competence, the ways they used digital technologies in their role as school leaders, and the opportunities and challenges they felt digital technologies brought to the task of leading a school. Their contributions strongly focussed on digital technology regarding teachers and their teaching practice. Because school leaders’ digital competence affects the organisation of digital technologies in education, affecting equality within and among schools, this aspect requires future research. In addition, school leaders’ understanding of the digitalisation process in K–12 schools and their role as school leaders requires future research. Future research could involve a deeper study of school leaders’ practice for leading the digitalisation process in K–12 schools. Further, more research on school leaders’ need for digital competence to support teachers’ application of digital technologies in teaching is needed. Even if the study has been made in the Swedish context, digital technologies are increasing worldwide, and leadership is needed to organise digital technologies, making this contribution interesting for other geographical contexts. However, other contexts may differ with different backgrounds, conditions, opportunities, challenges, and needs, which is important to consider.

## Data Availability

Upon request, the corresponding author can provide the data analysed during the current study.

## References

[CR1] A’mar F, Eleyan D (2022). Effect of principal’s technology leadership on teacher’s technology integration. International Journal of Instruction.

[CR2] Agélii Genlott A, Grönlund Å, Viberg O (2019). Disseminating digital innovation in school–leading second-order educational change. Education and Information Technologies.

[CR3] Agélii Genlott, A., Grönlund, Å., Viberg, O., & Andersson, A. (2021). Leading dissemination of digital, science-based innovation in school – A case study. *Interactive Learning Environments.*10.1080/10494820.2021.1955272

[CR4] AlAjmi MK (2022). The impact of digital leadership on teachers’ technology integration during the COVID-19 pandemic in Kuwait. International Journal of Educational Research.

[CR5] Braun V, Clarke V (2006). Using thematic analysis in psychology. Qualitative Research in Psychology.

[CR6] Braun V, Clarke V (2021). Thematic analysis: A practical guide.

[CR7] Christensen R, Eichhorn K, Prestridge S, Petko D, Sligte H, Baker R, Alayyar G, Knezek G (2018). Supporting learning leaders for the effective integration of technology into schools. Technology, Knowledge and Learning.

[CR8] Cohen L, Manion L, Morrison K (2011). Research methods in education.

[CR9] Conrads, J., Rasmussen, M., Winters, N., Geniet, A., & Langer, L. (2017). Digital education policies in Europe and beyond: Key design principles for more effective policies. *European Commission, Joint Research Centre.*10.2760/462941

[CR10] Corlatean T (2020). Risks, discrimination and opportunities for education during the times of COVID-19 pandemic. Research Association for Interdisciplinary Studies.

[CR11] Ewing L-A, Cooper HB (2021). Technology-enabled remote learning during COVID-19: Perspectives of Australian teachers, students and parents. Technology, Pedagogy and Education.

[CR12] Flanagan L, Jacobsen M (2003). Technology leadership for the twenty-first century principal. Journal of Educational Administration.

[CR13] Fullan M (2015). The new meaning of educational change.

[CR14] Gu L, Lindberg OJ, Krejsler JB, Moos L (2021). Understanding Swedish educational policy developments in the field of digital education. What works in Nordic school policies?: Mapping approaches to evidence, social technologies and transnational influences.

[CR15] Haelermans C (2017). Digital tools in education on usage, effects, and the role of the teacher.

[CR16] Håkansson Lindqvist M (2019). School leaders’ practices for innovative use of digital technologies in schools. British Journal of Educational Technology.

[CR17] Håkansson Lindqvist M, Pettersson F (2019). Digitalization and school leadership: On the complexity of leading for digitalization in school. The International Journal of Information and Learning Technology.

[CR18] Hauge TE, Norenes SO, Vedøy G (2014). School leadership and educational change: Tools and practices in shared school leadership development. Journal of Educational Change.

[CR19] Hopkins D (2017). The William Walker oration. The past, present and future of school improvement and system reform.

[CR20] Howard SK, Tondeur J, Siddiq F, Scherer R (2021). Ready, set, go! Profiling teachers’ readiness for online teaching in secondary education. Technology, Pedagogy and Education.

[CR21] Ittner D, Hagenauer G, Hascher T (2019). Swiss principals’ emotions, basic needs satisfaction and readiness for change during curriculum reform. Journal of Educational Change.

[CR22] Jaldemark J, Håkansson Lindqvist M, Mozelius P, Ryberg T (2021). Editorial introduction: Lifelong learning in the digital era. British Journal of Educational Technology.

[CR23] Jandrić P, Hayes D, Truelove I, Levinson P, Mayo P, Ryberg T, Monzó LD, Allen Q, Stewart PA, Carr PR (2020). Teaching in the age of Covid-19. Postdigital Science and Education.

[CR24] Jandrić P, Martinez AF, Reitz C, Jackson L, Grauslund D, Hayes D, Lukoko HO, Hogan M, Mozelius P, Arantes JA (2022). Teaching in the age of Covid-19—The new normal. Postdigital Science and Education.

[CR25] Kemmis S, Wilkinson J, Edwards-Groves C, Hardy I, Grootenboer P, Bristol L (2014). Changing practices, changing education.

[CR26] Khlaif ZN, Salha S, Affouneh S, Rashed H, ElKimishy LA (2021). The Covid-19 epidemic: Teachers’ responses to school closure in developing countries. Technology, Pedagogy and Education.

[CR27] Larsson P, Löwstedt J (2020). Strategier och förändringsmyter: Ett organisationsperspektiv på skolutveckling och lärares arbete [strategies and change myths: An organizational perspective on school development and teachers’ work].

[CR28] Leithwood K, Harris A, Hopkins D (2019). Seven strong claims about successful school leadership revisited. School Leadership & Management.

[CR29] Liljenberg M (2015). Distributing leadership to establish developing and learning school organisations in the Swedish context. Educational Management Administration & Leadership.

[CR30] Masters J (2018). Trends in the digitalization of K–12 schools: The Australian perspective. Seminar.net.

[CR31] Mingaine L (2013). Leadership challenges in the implementation of Ict in public secondary schools. Kenya. Journal of Education and Learning.

[CR32] OECD. (2021). *Pisa 2021 ICT Framework*. https://www.oecd.org/pisa/sitedocument/PISA-2021-ICT-Framework.pdf

[CR33] Olofsson AD, Fransson G, Lindberg JO (2020). A study of the use of digital technology and its conditions with a view to understanding what ‘adequate digital competence’may mean in a national policy initiative. Educational Studies.

[CR34] Pettersson F (2018). On the issues of digital competence in educational contexts – A review of literature. Education and Information Technologies.

[CR35] Pettersson F (2021). Understanding digitalization and educational change in school by means of activity theory and the levels of learning concept. Education and Information Technologies.

[CR36] Raman A, Thannimalai R, Ismail SN (2019). Principals’ technology leadership and its effect on teachers’ technology integration in 21st century classrooms. International Journal of Instruction.

[CR37] Reis-Andersson J (2023). School organisers’ expression on the expansion of the access and application of digital technologies in educational systems. The International Journal of Information and Learning Technology.

[CR38] Selwyn, N., Nemorin, S., Bulfin, S., & Johnson, N. F. (2018). Everyday schooling in the digital age. *Routledge.*10.4324/9781315115764

[CR39] Swedish Government. (2017). *Nationell digitaliseringsstrategi för skolväsendet [National digitalisation strategy for the school system]*. https://www.regeringen.se/4a9d9a/contentassets/00b3d9118b0144f6bb95302f3e08d11c/nationell-digitaliseringsstrategi-for-skolvasendet.pdf

[CR40] Tondeur J, Van Braak J, Ertmer PA, Ottenbreit-Leftwich A (2017). Understanding the relationship between teachers’ pedagogical beliefs and technology use in education: A systematic review of qualitative evidence. Educational Technology Research and Development.

[CR41] Uzorka A, Olaniyan AO (2022). Leadership role and professional development of technology. Education and Information Technologies.

[CR42] Viberg O, Mavroudi A, Khalil M, Bälter O (2020). Validating an instrument to measure teachers’ preparedness to use digital technology in their teaching. Nordic Journal of Digital Literacy.

[CR43] Woodcock S, Hardy I (2022). ‘You’re probably going to catch me out here’: Principals’ understandings of inclusion policy in complex times. International Journal of Inclusive Education.

